# The Electrode Modality Development in Pulsed Electric Field Treatment Facilitates Biocellular Mechanism Study and Improves Cancer Ablation Efficacy

**DOI:** 10.1155/2017/3624613

**Published:** 2017-05-07

**Authors:** Chao Cen, Xinhua Chen

**Affiliations:** ^1^The Key Laboratory of Combined Multi-Organ Transplantation, Ministry of Public Health and The Department of Hepatobiliary and Pancreatic Surgery, The First Affiliated Hospital, School of Medicine, Zhejiang University, Hangzhou 310003, China; ^2^The Collaborative Innovation Center for Diagnosis and Treatment of Infectious Diseases, The First Affiliated Hospital, School of Medicine, Zhejiang University, Hangzhou 310003, China

## Abstract

Pulsed electric field treatment is now widely used in diverse biological and medical applications: gene delivery, electrochemotherapy, and cancer therapy. This minimally invasive technique has several advantages over traditional ablation techniques, such as nonthermal elimination and blood vessel spare effect. Different electrodes are subsequently developed for a specific treatment purpose. Here, we provide a systematic review of electrode modality development in pulsed electric field treatment. For electrodes invented for experiment in vitro, sheet electrode and electrode cuvette, electrodes with high-speed fluorescence imaging system, electrodes with patch-clamp, and electrodes with confocal laser scanning microscopy are introduced. For electrodes invented for experiment in vivo, monopolar electrodes, five-needle array electrodes, single-needle bipolar electrode, parallel plate electrodes, and suction electrode are introduced. The pulsed electric field provides a promising treatment for cancer.

## 1. Introduction

In recent years, pulsed electric field treatment has been gaining extensive attention in virtue of its biological and medical applications, such as gene delivery [1–5], electrochemotherapy [6–11], and cancer therapy [12–18]. One advantage of pulsed electric field treatment, making it distinctive from other physical techniques, is the ability to destroy tissues or tumors in a nonthermal manner [19, 20]. Consequently, pulsed electric field treatment makes it possible to preserve sensitive tissues intact, such as blood vessels and axons [21, 22]. Furthermore, this minimally invasive technique allows the possibility of regeneration with healthy cells and tissues in the treatment region and leaves a minimal scar [23]. With the aid of ultrasound, CT, or MRI, pulsed electric field treatment could be monitored in real time, which helps improve the treatment efficiency immensely [24–26].

Conventional appliance consists of three parts: pulse generator, electrodes, and connection links between them. The pulse generator produces square wave pulses at regular intervals. Amplitude, pulse width, period, and phase delay are the primary parameters to determine the shape of the output waveform. Electric field strength, depending on the amplitude of the pulse and the distance between the electrodes, is often crucial for completed treatment effect [27]. When electrodes are unsuitable, the strength in a certain target area is insufficient, resulting in incomplete treatment effects. As the literature focusing on this research field is scarce, an overview of electrodes appears very timely. This review can be used to improve electrodes for individual-based treatment and guide new electrode designs.

We incorporate papers that are representative of existing technology and indicative of future directions, based on the massive amount of literature about electrode assemblies used in pulsed electric field treatment. As current medical applications of pulsed electric fields are mainly concentrated on cell and tissue treatment, this review is divided into two sections: section A, electrodes invented for in vitro (cell response to the treatment), and section B, electrodes invented for in vivo (tissue response to the treatment).

## 2. Part I: Electrode Invented for Experiment In Vitro

### 2.1. Traditional Electrodes for Membrane Charging Measurement

**Figure 1:**
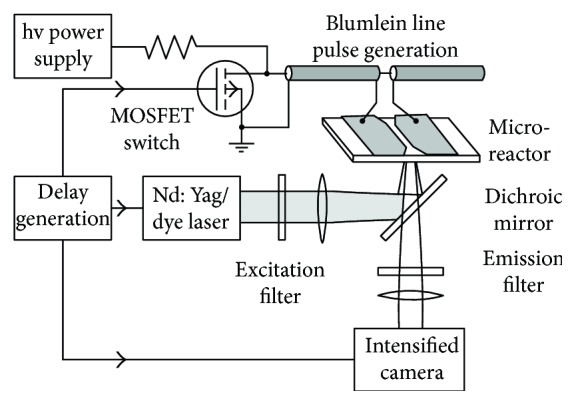
Traditional electrodes assembled with dye laser (cited from [28], Figure 1).

A cell is often described as a conductive body (the cytoplasm) surrounded by a dielectric layer (the surface membrane). When a cell is applied to an electric field in a conductive medium, electric charges accumulate at the cell membrane and consequently form a voltage across the membrane. Frey et al. [28] designed a system (Figure 1) to investigate plasma membrane voltage changes in response to nanosecond pulsed electric fields. Jurkat cells were stained with Annine-6 (a novel voltage-sensitive hemicyanine dye with a subnanosecond temporal response, optically measuring the changes in transmembrane voltage of excitable cells [29]) and then exposed to an electric field of 95 kV/cm for 60 ns. Then, the illumination was provided by a dye laser whose wavelength was close to the excitation maximum of Annine-6 and pulse duration was far less than the duration of nanosecond pulsed electric field exposure. Taking the advantage of the technical progress, membrane voltage changes with time were monitored. The results showed a strong asymmetry between the anodic and cathodic poles: the membrane facing the anodic pole reached values of 1.6 V after 15 ns, compared with only 0.6 V at the cathodic pole at the same time (figure not shown). These facilities make it possible to monitor the real-time cell membrane voltage changes in site so that the firsthand direct cell response can be recorded objectively.

Another nonignorable conclusion drawn from this electrode model is intracellular effect of nanosecond pulsed electric fields—almost all the voltage was applied across the interior of the cell. This phenomenon has been confirmed by several studies [30–34]. Among all the complex cascade of events, including chromatin condensation and nucleosomal DNA fragmentation, disruption of mitochondrial membrane potential is often considered to be involved in activation of caspase-mediated apoptosis. Thus, with this electrode model, the mitochondrial membrane potential can be also detected with fluorescent dye rhodamine 123 [35], TMRE [36], DiOC_6_ [3], JC-1, and TMRM.

When plasma membrane voltage exceeds a critical value (about 500 mV), transmembrane pores form, which is known as electroporation [37]. It is found that the pore size is a function of the duration of the pulse. Microsecond pulses generate large pores (conventional electroporation), which are big enough to deliver macromolecules, such as DNA [1–5], dyes [38], and drugs [39]. In contrast, the pores generated by a nanosecond pulsed electric field are only about 1 nanometer wide, which are often named as “nanopores.” Nanopores have little permeability to propidium iodide (a macromolecule dye used to stain DNA) [40] but show permeability to small inorganic ions, distinguishing from conventional electroporation. This hypothesis was supported by a series of researches, cellular uptake of Tl^3+^ [41], Cu^2+^ [42], and Ca^2+^ [43, 44], among which cellular uptake of Ca^2+^ is mostly related to biological functions. Increased intracellular Ca^2+^ may be involved in triggering mitochondrial apoptosis pathway via cytochrome c [45–48].

### 2.2. Sheet Electrode and Electrode Cuvette

**Figure 2:**
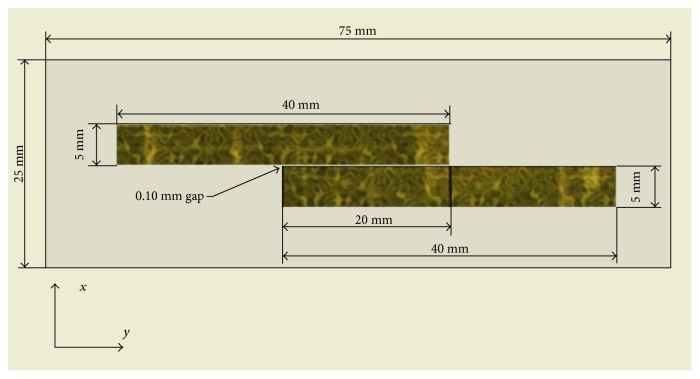
Sheet electrode (cited from [50], Figure 1).

**Figure 3:**
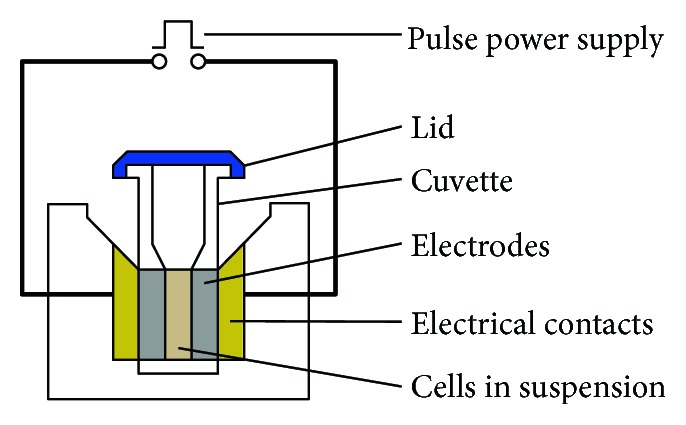
Electroporation cuvette (cited from http://commons.wikimedia.org).

Several previous studies have been designed to figure out whether Ca^2+^ comes from intracellular Ca^2+^ pools or extracellular solutions. Vernier et al. [49] demonstrated in 2003 that nsPEF triggers Ca^2+^ release from the endoplasmic reticulum in Jurkat cells, and White et al. [40] confirmed it in HL-60 cells in 2004. However, Craviso et al. [50] did not find Ca^2+^ release from the endoplasmic reticulum using adrenal chromaffin cells. They fabricated microelectrode chambers on a glass microscope slide with gold electrodes (Figure 2). With the indication of the calcium-sensitive fluorescence indicator Calcium Green, the intracellular calcium level of adrenal chromaffin cells could be detected. Consistent with previous findings, the rise of intracellular calcium depends on extracellular calcium. In parallel experiments, chromaffin cells were transferred to electroporation cuvettes (Figure 3) for nanosecond pulsed electric field exposure. The release of norepinephrine and epinephrine was determined by high-performance liquid chromatography coupled with electrochemical detection. It was concluded that nanosecond pulsed electric field could elicit calcium-dependent catecholamine release in chromaffin cells.

Sheet electrodes are characterized by two parallel pieces of gold foil (5 mm wide; 25 *μ*m thick; 100 *μ*m apart), placed partly overlapped. This design ensures a homogeneous electric field and maximally eliminates “fringing effects” due to the sharp edges of the microchamber electrodes. When a large number of cells need to be treated, the electroporation cuvette (1 mm electrode) may be the first choice. It could contain nearly 10^6^ cells in a 200 *μ*l suspension and provide a homogeneous electric field, but “fringing effects” seems hard to avoid.

### 2.3. The Cylinder Tungsten Electrodes with High-Speed Fluorescence Imaging System

**Figure 4:**
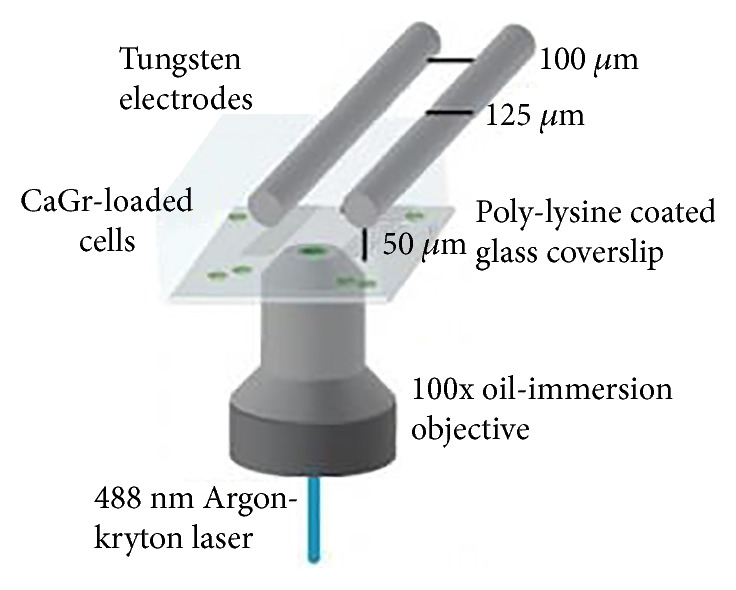
The tungsten electrodes with high-speed fluorescence imaging system (cited from [43], Figure 1).

As stated above, Craviso et al. put forward that intracellular Ca^2+^ primarily came from extracellular pools, but the detection precision was limited to 1 second. To find out what happens in the first 1 second, Beier et al. [43] set up a high-speed fluorescence imaging system (Figure 4) to monitor Ca^2+^ movement and proposed the opposite opinion: intracellular calcium pools seem much important. Rodent neuroblastoma cells were cultured on a glass-bottom poly-L-lysine coated dish and incubated in exposure buffer for 30 min prior to nanosecond pulsed electric field, avoiding interference of changes of buffer solution. An argon-krypton ion laser tuned to 488 nm was employed to excite the intracellular florescent dye. Different conventional imaging methods, such as epifluorescence and confocal microscopy [51], are too slow to capture the possibly instant Ca^2+^ influx. Timing of the imaging system, laser irradiation, and nanosecond pulsed electric field delivery was controlled by using a digital delay generator (Stanford Research Systems), making it feasible to record Ca^2+^ influx in milliseconds. The results showed that an increased Ca^2+^ concentration was visible after 3.5 ms. In addition, it provides evidence that intracellular Ca^2+^ concentration increases in the absence of extracellular calcium; alluding intracellular calcium pools play a major role in intracellular Ca^2+^ concentration increase when exposed to nanosecond pulsed electric field. These results benefit mostly from the utilization of high-speed fluorescence imaging system and visualize the Ca^2+^ in milliseconds. Taking advantage of this appliance, other micromolecules can be detected in the very early stage such as Tl^3+^ and Cu^2+^.

### 2.4. Tungsten Electrodes in Combination with Patch-Clamp

**Figure 5:**
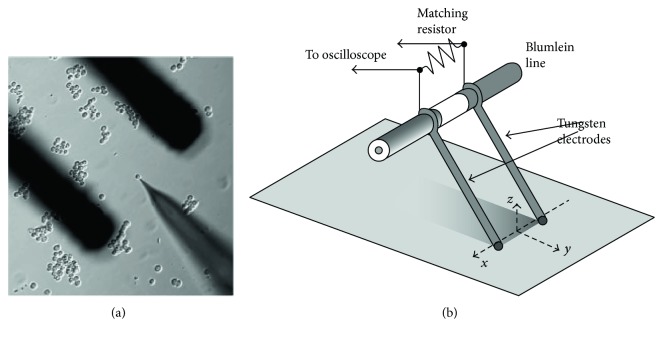
The tungsten electrodes in combination with patch-clamp (cited from [52], Figure 1).

Another important question is how long the nanopores last. Patch-clamp was employed to explore nanopores on cell membrane and provide new insights [52]. Cells were suspended on a cover slip and then tungsten electrodes were positioned with a micromanipulator at the sides of the cell (Figure 5()). To avoid the interference of patch-clamp to the cell exposed to nanosecond pulsed electric field, patch pipette keeps intact with the cell until 50 s (80–120 s on average) after the exposure. Even with such a long delay, profound decrease of cell membrane resistance (*R*_m_) was still detected, accompanied by the loss of the membrane potential. The early studies estimated that pulsed electric fields cause cell membrane permeabilization opening big pores in cell membrane, but currently, patch clamp technology revealed that ultrashort intensive pulsed electrictric field opens small plasma membrane pores [33, 49, 53]. Similar to a conventional electrodes design, tungsten electrodes generated homogeneous electric fields. This new finding mainly benefits from the application of patch-clamp, which could detect electric current through the nanopores in a much smaller order of magnitude. Patch-clamp provides a precise approach to measure single or multiple ion channel responses to nanosecond pulsed electric field treatment. Some works carrying on demonstrate that nanosecond pulsed electric field may have the effect of not only long-term permeabilization but also inhibition of voltage-gated *I*_Na_ and *I*_Ca_ [54, 55].

### 2.5. Electrodes Assembled with Confocal Laser Scanning Microscopy

**Figure 6:**
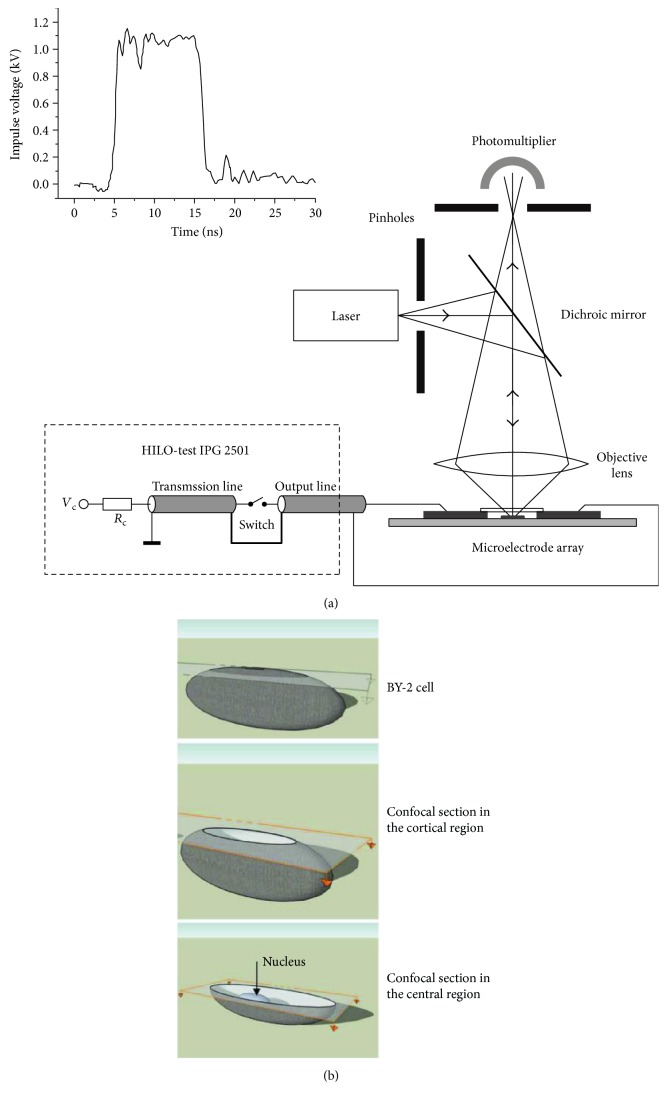
Electrodes assembled with confocal laser scanning microscopy (cited from [51], Figure 1).

As mentioned in Section 2.1, nanosecond pulsed electric field can penetrate into the cell interior, disturbing mitochondrial membrane potential. However, experiments visualizing the internal membrane to monitor the real-time response to the nanosecond pulsed electric field are rare. Confocal laser scanning microscopy is often deemed to be competent to view subcellular structures. Berghöfer et al. then designed confocal microscopy to the nanosecond pulsed electric field system [51]. Tobacco wild cell line (BY-2) was used that expressed GFP in fusion with markers for tubulin, endoplasmic reticulum, and actin filaments. As seen in Figure 6, confocal laser scanning microscopy was assembled to conventional nanosecond pulsed electric field treatment system, and interior structures in selected depths were imaged in high-resolution. Microtubules disorder, actin disassembly, and nuclear envelope disintegration were observed; insinuating nanosecond pulsed electric field affects not only the internal membrane but also cytoskeleton structures. Confocal microscopy is featured for its ability to acquire in-focus images from selected depths. Images are acquired point by point and reconstructed with a computer, allowing three-dimensional reconstructions of the cell. So, this invention provides a method to view subcellular structures responses to nanosecond pulsed electric field in three-dimension format.

## 3. Part II: Electrode Invented for Experiment In Vivo

Beside resection or transplantation surgery, the focal ablation techniques such as cryosurgery, microwave (MW), laser interstitial thermal therapy (LITT), high-intensity focused ultrasound (HIFU), and radiofrequency ablation (RFA) remain to be fundamental means for cancer treatment. Cryosurgery freezes the tissue as low as −40°C to cause tissue necrosis. MW, LITT, HIFU, and RFA often heat the tissue to be more than 60°C, producing cell death and coagulative necrosis [56]. However, these thermal techniques cause inevitable complications, such as incomplete treatment of vascularized tissue (“sink effect” of the blood), possible damage to the blood vessel [57], production of unexpected scar [58], and exclusion of tumor lying within 1 cm of the overlying skin for fear of burning of the skin or muscle [59]. As a nonthermal technique, nanosecond pulsed electric field ablation has a better performance in avoiding these thermal complications, which raises the tissue temperature by only 0.3°C [60].

### 3.1. Traditional Monopolar Electrodes

**Figure 7:**
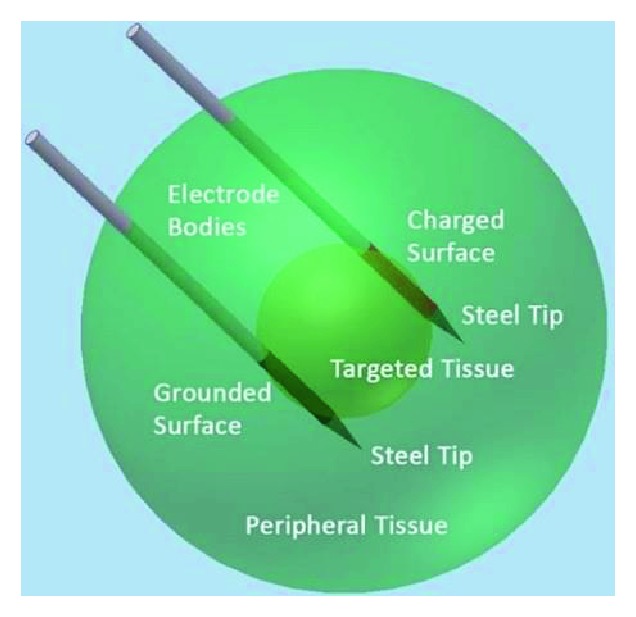
The monopolar electrodes (cited from [61], Figure 1).

The prevailing electrodes used for the treatment of solid cancer consist of two needles, one is charged and the other is grounded (as shown in Figure 7). The electrodes were chosen to be 1 mm in diameter and separated 0.8 cm apart [61]. The advantage of monopolar electrodes is the flexibility for electrode layout to optimize treatment of a targeted region, satisfying the requirements of individual-based treatment. In addition, monopolar electrodes are often used as a standard model to measure the varying strength around the needle in space.

### 3.2. Five-Needle Array Electrodes

**Figure 8:**
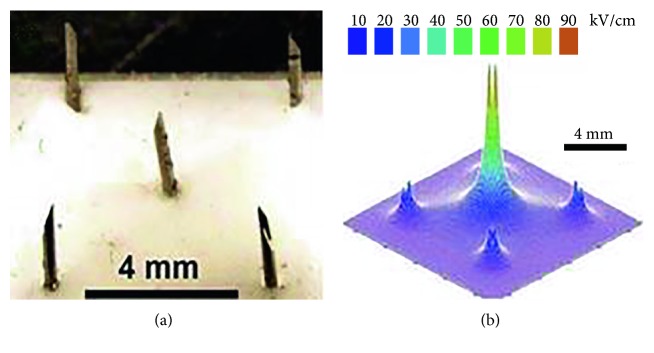
Needle array electrode and electric field pattern (cited from [62], Figure 2).

To enlarge the treatment area, five-needle array was applied [62]. The needle array (Figure 8) was made using 30 gauge hypodermic needles (300 *μ*m diameter) extending 2 mm from a Teflon base. The center needle forms the anode; the other four surrounding needles spaced 4 mm from the center electrode form the cathode. To avoid flashover between needles and accompanying skin damage, skin was coated with vegetable oil. Melanomas shrinks to about 10% of original size after the first treatment and complete remission is observed after the second treatment. The effect of 5-needle array was also confirmed in another study [63] in which bioelectrical field ablation efficiently eliminated papillomas and squamous cell carcinoma in vivo from skin of mice (figure not shown). However, the tumor treated has to be fit for the size of five-needle array electrodes, which hinders the popularization in other areas.

### 3.3. Single-Needle Bipolar Electrode

**Figure 9:**
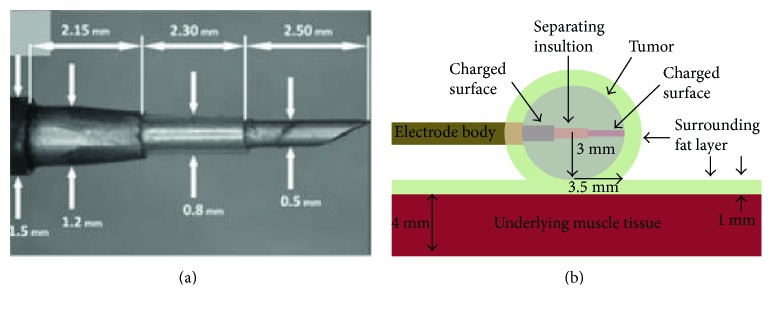
The single-needle bipolar electrode (cited from [64], Figure 1).

Although the larger electrode with multiple needles could cause enlarged electric field coverage, problems such as placement complexity and more physical damage from electrode penetration come up consequently. So, a single needle bipolar electrode was used [64]. The electrode is divided into two parts: an electrode body coated with an insulating layer and a tip containing two electrically conductive surfaces separated by an additional insulating layer (Figure 9). To detect the effects of this single needle bipolar electrode, bioluminescent images and histological examination were performed. The results showed that the treatment achieved breast cancer regression in mice. Featured for its convenience, single-needle bipolar electrode generates an inhomogenous electric field, which means electric field may not cover the tumor completely. Operators have to apply more pulse times to ensure the complete elimination. Taking advantage of its convenience, if this single needle bipolar electrode could be further improved, it will be a promising alternative of bioelectrical field ablation.

### 3.4. The Noninvasive Parallel Plate Electrodes

**Figure 10:**
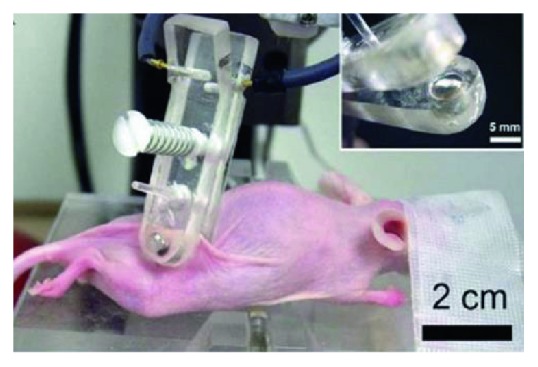
Photograph of noninvasive parallel plate electrodes (cited from [62], Figure 6).

As techniques improve, noninvasive surgery and instruments are in great need. The noninvasive parallel plate electrodes were designed by Nuccitelli et al. [62]. The parallel plate electrodes (Figure 10) were made from stainless steel, and the size could be adjusted according to the treated tumor from 3 mm to 5 mm in diameter. Each tumor was placed between two plates with a separation of 0.5–1 mm. A thick layer of conductive agar coated the electrodes to separate skin from the electrodes. Tumor shrank in a large extent in spite of no elimination. A black scab appeared on the stratum corneum as the skin was exposed to the electric pulse fields. The main advantage of parallel plate electrodes is that they could deliver a more equally distributed electric field and ensure the target area is exposed to the similar electric field strength.

### 3.5. Suction Electrode

Similar to parallel plate electrodes, Nuccitelli et al. designed suction electrodes (Figure 1()) for noninvasive treatment [60, 65]. Suction electrodes are plastic cylinders with a cup-shaped opening at one end with an inner diameter of 4 mm and a depth of 2 mm. At the base of each cup were small holes, allowing a suction to be applied to pull the tumor into the cavity. The treatment area was highly localized to the tissues in the suction electrodes. The suction removes air from the cavity, then gases are not available for ionization by the high electric fields applied that could lead to arcing. Similar to five-needle array electrodes, the limitation was that treatment size was subjected to the “electrode size” and the treatment area was limited to the subcutaneous tissue.

### 3.6. Electrodes Invented for Brain Treatment

**Figure 12:**
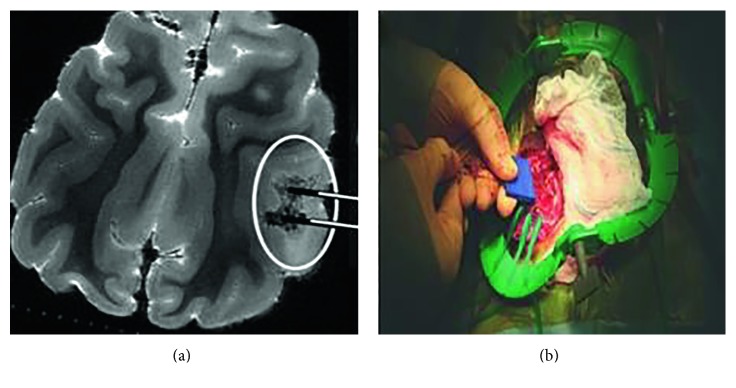
Electrode used in brain. (a) Cited from [27], Figure 1. (b) Cited from [70].

When applied to clinical application, pulsed electric field was initially used to destroy substantial volumes of tissue [63, 65–68], then it extended the application to liver [24, 60, 69], prostate [21], and sarcoma tumors [19]. Garcia et al. [27] provided a preliminary study of brain cancer therapy with conventional monopolar electrodes (Figure 12). The probes were 1 mm in diameter with an insulating sheath, where 0.5-cm-long tips were exposed in contact with the tissue. Combining with previous [25] and latter [70] relevant researches by this group, the results showed the safety of bioelectrical field ablation in the brain and the affected lesion volume was correlated with the applied voltage. Intracranial approach in neurosurgery is still in the initial stage, and this new invention has shown advantages superior to the invasive resection: (1) the small electrode size ensures the procedure minimally invasive and adaptable to almost any neuroanatomical location under the guidance of ultrasonography and MR imaging; (2) bioelectrical field ablation makes it feasible to create a sharply delineated volume of ablated tissue with submillimeter resolution in the brain, where tumors are often deep-seated and well-circumscribed; and (3) blood vessel spare effect maximally protects the sensitive tissues adjacent to the tumor.

## 4. Conclusion

Here, we provide a brief review of the electrode reported in the pulsed electric field treatments in vitro and in vivo. The specific applications, the advantages, and the disadvantages are also discussed. It was reported [71] that a first-in-human trial nanoelectroablation therapy for basal cell carcinoma has been recently carried out. The results confirmed the safety and feasibility of the pulsed electric field ablation. The electrodes are expected to attain the maximum efficiency and minimal complications.

## Figures and Tables

**Figure 11:**
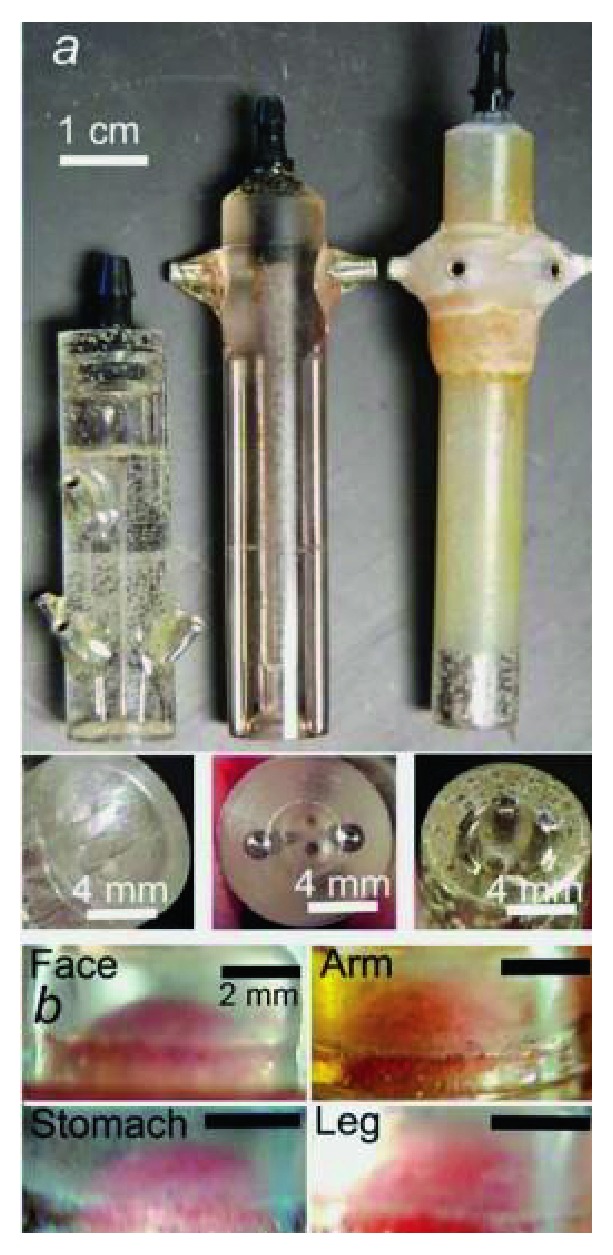
Suction electrodes (cited from [65], Figure 1).

## Abbreviations

CT: Computed tomography
DiOC_6_: 3,3′-Dihexyloxacarbocyanine iodide
DNA: Deoxyribonucleic acid
GFP: Green fluorescent protein
HIFU: High-intensity focused ultrasound
JC-1: 5,5′,6,6′-Tetrachloro-1,1′,3,3′-tetraethylbenzimidazolylcarbocyanine iodide
LITT: Laser interstitial thermal therapy
MRI: Magnetic resonance imaging
MW: Microwave
RFA: Radiofrequency ablation
*R*_m_: Cell membrane resistance
TMRM: Tetramethylrhodamine.
